# RETRACTION: Clinical Study on Orthopaedic Treatment of Chronic Osteomyelitis With Soft Tissue Defect In Adults

**DOI:** 10.1111/iwj.70666

**Published:** 2025-04-23

## Abstract

**RETRACTION:** ZhangX. , YangX. , ChenY. , WangG. , SingP. , ZhaoZ. , and BiH. , “Clinical Study on Orthopaedic Treatment of Chronic Osteomyelitis With Soft Tissue Defect In Adults,” International Wound Journal 19, no. 6 (2022): 1349–1356, 10.1111/iwj.13729.34935287 PMC9493237

The above article, published online on 21 December 2021, in Wiley Online Library (http://onlinelibrary.wiley.com/), has been retracted by agreement between the journal Editor in Chief, Professor Keith Harding; and John Wiley & Sons Ltd. Following an investigation by the publisher, all parties have concluded that this article was accepted solely on the basis of a compromised peer review process. The editors have therefore decided to retract the article. The authors did not respond to our notice regarding the retraction.

**RETRACTION:** X. Zhang , X. Yang , Y. Chen , G. Wang , P. Sing , Z. Zhao , and H. Bi , “Clinical Study on Orthopaedic Treatment of Chronic Osteomyelitis With Soft Tissue Defect In Adults,” International Wound Journal 19, no. 6 (2022): 1349–1356, 10.1111/iwj.13729.34935287 PMC9493237

The above article, published online on 21 December 2021, in Wiley Online Library (http://onlinelibrary.wiley.com/), has been retracted by agreement between the journal Editor in Chief, Professor Keith Harding; and John Wiley & Sons Ltd. Following an investigation by the publisher, all parties have concluded that this article was accepted solely on the basis of a compromised peer review process. The editors have therefore decided to retract the article. The authors did not respond to our notice regarding the retraction.

